# The role of superb microvascular imaging in detecting low-grade inflammation among adults and those with chronic kidney disease: A preliminary study

**DOI:** 10.55730/1300-0144.5632

**Published:** 2022-12-03

**Authors:** Alaaddin NAYMAN, Nusret SEHER, Mehmet ÖZTÜRK, Halil ÖZER, Gülperi ÇELİK

**Affiliations:** 1 Department of Radiology, Faculty of Medicine, Selçuk University, Konya, Turkey; 2 Department of Internal Medicine Sciences, Faculty of Medicine, Selçuk University, Konya, Turkey

**Keywords:** Inflammation, superb microvascular imaging, adult, chronic kidney disease, muscle wasting disease

## Abstract

**Background/aim:**

Pathophysiologic changes associated with chronic inflammation occur with aging and more prominently in patients with chronic kidney disease (CKD), and an association between chronic inflammation and muscle wasting has been identified. The microcirculation is extremely sensitive to the inflammatory process and actively participates in it. In a healthy adult, angiogenesis is a strictly controlled and rare occurrence. However, aberrant angiogenesis and the development of new tiny blood vessels are known in chronic inflammatory diseases. Superb microvascular imaging (SMI) is a noninvasive technique that can evaluate tiny vessels with low blood flow and provide quantitative data. Our goal was to detect increased blood flow secondary to low-grade chronic inflammation in micro-circulation in the rectus femoris (RF) muscle using SMI.

**Materials and methods:**

This cross-sectional study involved 30 patients with CKD, 30 adults without CKD or other chronic illnesses, and 32 young healthy volunteers. This study was conducted in our university hospital between March and December 2021. The RF cross-sectional area (CSA) was measured, and vascular index (VI) values were obtained using SMI. All three groups’ RF-CSA and VI values were compared.

**Results:**

Although there was no statistically significant difference in RF-CSAs between the groups, the VI values of all three groups were statistically different (p < 0.001). The median (min–max) VI values were 0.90 (0.60–1.30), 0.50 (0.20–1.0), and 0.30 (0.10–0.50) for the CKD, adult control, and young healthy groups, respectively. The VI significantly differentiated patients with CKD from all other patients and the adult control group. When a cutoff value of 6.5 was used for the VI in detecting increased blood supply in RF muscle in patients with CKD, the accuracy, positive predictive value, and negative predictive value were 93.5%, 85.3%, and 98.3%, respectively.

**Conclusion:**

SMI can detect increased blood supply caused by low-grade inflammation in the RF muscle.

## 1. Introduction

Muscle wasting diseases (MWD), such as sarcopenia, are currently a hot research topic, due to increased awareness of the increasing prevalence of such conditions based on population aging [1,2]. Rosenberg coined the term sarcopenia in 1989 to define the reduction of skeletal muscle mass and strength with age [3]. Chronic kidney disease (CKD) causes an energy imbalance due to decreased protein intake and accelerated catabolism. As a result, sarcopenia may occur in patients with CKD during young adulthood [4,5]. Studies have shown that low muscle strength resulting from sarcopenia is associated with impaired clinical outcomes, diminished life quality, increased hospitalization, and increased mortality [4]. Several chronic diseases are associated with muscle mass loss among older adults, and whether this is due to aging or disease is unknown [6]. On the other hand, inflammation is a common feature of chronic disease (with or without aging), and inflammation is frequently associated with hyperemia [7–9]. Inflammation is also associated with an increase in blood vessel proliferation [10].

Ultrasound (US) has been a reliable tool for determining muscle mass and quality by measuring the cross-sectional area (CSA), thickness, and volume of muscle [11]. Ultrasonographic elastography and contrast-enhanced US can detect muscle stiffness and changes in microvascular structure caused by sarcopenia; however, data are still limited [12,13]. US is commonly used in musculoskeletal (MSK) disorders due to advantages such as low cost and easy accessibility. However, there is no standardized threshold value for US measurements used to quantify muscle mass and quality, which significantly limits its applicability in sarcopenia [1]. It is well known that the capacity of Doppler US (DUS) to identify pathologic flow within MSK soft tissue indicates the presence of local active inflammation [14]. However, conventional DUS techniques have technical limitations in imaging small blood vessels due to exogenous Doppler signals caused by clutter such as surrounding tissue motion. Accordingly, wall filters should be used to eliminate clutter and motion distortions from conventional DUS imaging. This leads to reduced visualization of small blood vessels with slower blood flow that is approximately equivalent to the speed of tissue movement [15]. These limitations have been resolved by advanced flow detection imaging technology.

Superb microvascular imaging (SMI) is a novel technology that can separate flow data from overlapping tissue movement distortions, retaining tiny slow-flowing vasculature with excellent clarity and detail. SMI analyzes clutter motion and employs a unique algorithm to determine and reduce tissue movement, expose genuine blood flow, and evaluate tiny vessels with blood flow at quite a low speed. SMI has been considered a novel method for assessing various MSK conditions, including lateral epicondylosis, arthritides, and carpal tunnel syndrome [16,17]. SMI can also obtain quantitative data known as the vascular index (VI). To the best of our knowledge, no study on low-grade inflammation in the rectus femoris (RF) muscle using SMI has yet been published.

Even though MWDs impose personal, social, and economic burdens if left untreated, early detection and initiation of necessary treatments are critical [6]. The goal of this study was to determine whether SMI could be used to detect low-grade muscle inflammation by finding increased blood supply in the RF muscle of adults and people with CKD.

## 2. Materials and methods

Following approval from the local ethics committee (2019/343), this cross-sectional study was conducted between March and December 2021. The study population was selected from patients who presented to internal medicine and ultrasound outpatient clinics. The US examinations were performed after all patients had been informed about the examinations and the procedure and their written consent had been obtained. The patients were categorized into three groups for evaluation. The first group included patients with CKD on hemodialysis, the second group included adult patients without CKD, and the third group comprised young healthy volunteers.

All evaluations were performed by a single radiologist (N.S.) who had 6 years of SMI experience using a DUS device (Canon Medical Systems, Tokyo, Japan) with a high-frequency (14 MHz) linear array transducer. The transducer was positioned three-fifths of the distance between the anterior superior iliac spine and the superior patellar border, perpendicular to the thigh’s long axis in its upper part [18]. This was the highest location of the thigh in all subjects at which the whole rectus femoris CSA (RF-CSA) could be demonstrated in a single field. Supine imaging was performed, with the resting leg supported in passive extension. To minimize underlying soft tissue diversion, excess contact gel was used.

**Figure 1:**
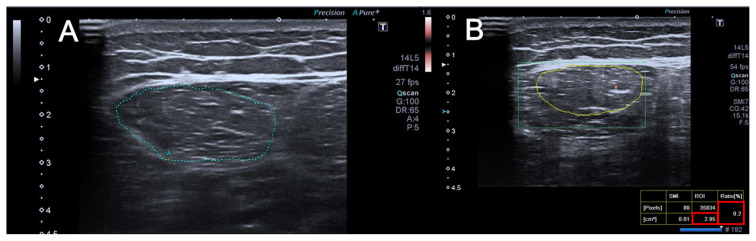
A 29-year-old healthy man. US (A) and SMI (B) images of the rectus femoris muscle are presented. The RF-CSA value is 2.95 cm^2^, and the VI value is 0.2.

**Figure 2:**
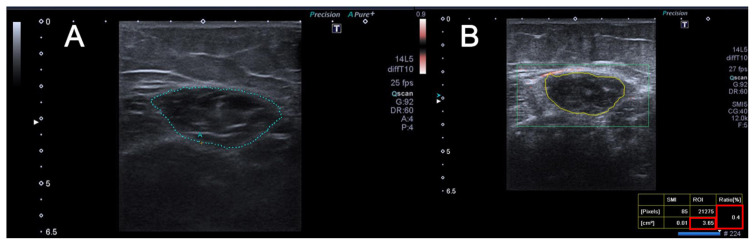
A 62-year-old woman without CKD or any other chronic disease. US (A) and SMI (B) images show the rectus femoris muscle. The RF-CSA value is 3.65 cm^2^, and the VI value is 0.4.

**Figure 3:**
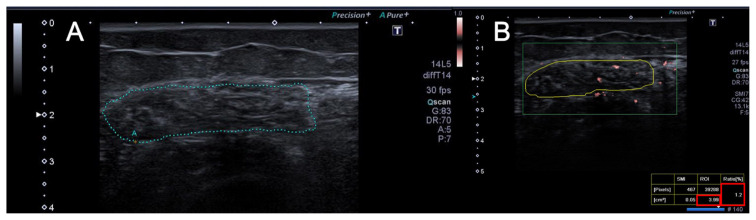
A 58-year-old man with CKD is on hemodialysis. US (A) and SMI (B) images of the rectus femoris muscle are presented. The RF-CSA value is 3.99 cm^2^, and the VI value is 1.2.

The internal echogenic line of the RF was manually outlined in a frozen image for RF-CSA calculation. The region of interest (ROI) was placed over the muscle to cover the entire muscle CSA, and quantitative data (VI values) were obtained through SMI examinations. Three measurements were retrieved and the average value was recorded (Figures 1A, 1B, 2A, 2B, 3A, and 3B). Images were obtained by selecting maximum Doppler gain and minimum pulse repetition frequency settings without allowing artifacts to occur because the vascular evaluation of the muscle is complex. All Doppler examinations in this study were performed using constant device parameters.

### 2.1. Statistical analysis

All procedures were conducted using the Statistical Package for the Social Sciences software (IBM SPSS Statistics 21.0, IBM Corporation, Armonk, NY, USA). The Shapiro-Wilk test was used to determine whether the scale variable distributions were normal. Descriptive statistics are reported as the mean and standard deviation for continuous numerical variables. Categorical variables are represented by the number of patients and percentages. The Kruskal-Wallis test and one-way analysis of variance (ANOVA) were used to compare continuous numerical data, and chi-square tests were used to compare categorical variables. For pairwise comparison of data sets, Bonferroni and Mann-Whitney U test post-hoc tests were performed. Receiver operating characteristic (ROC) curve analysis was used to evaluate the diagnostic performance. The optimal cut-off point was derived using the Youden index-if the area under the curve (AUC) was significant. DeLong’s test was used to compare the differences in AUC values. The sensitivity, specificity, positive predictive value (PPV), negative predictive value (NPV), and accuracy of diagnostic performance indicators were calculated. Differences were judged to be statistically significant at p < 0.05.

## 3. Results

In total, 92 patients (aged 17–93 years; 43 males, 49 females) were enrolled in this study, 30 of whom had CKD, 30 were adults without CKD or another chronic disease, and 32 were young healthy volunteers. There was no difference between the groups in terms of sex, body mass index, and RF-CSA (p > 0.05). The demographic features of the study population are presented in Table 1.

**Figure 4:**
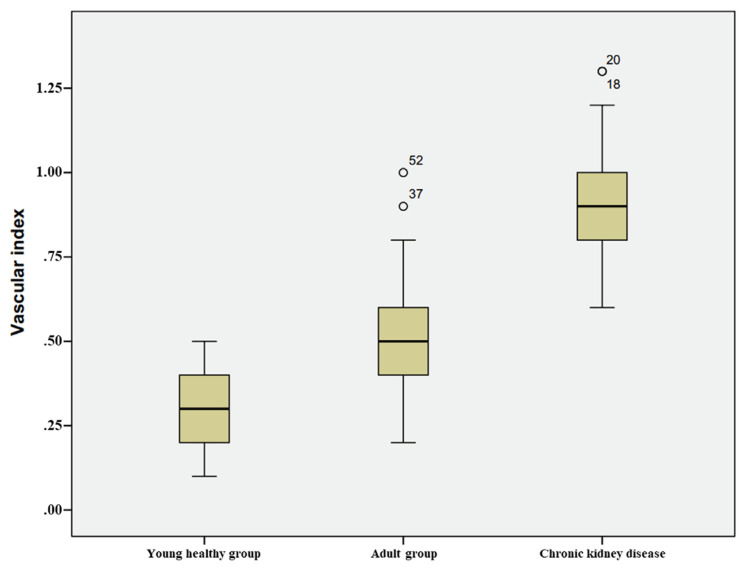
VI values of young, healthy volunteers, adult patients without CKD or another chronic disease, and CKD patients on hemodialysis. The values of all three groups are significantly different from each other.

The median (min-max) VI values were 0.90 (0.60–1.30), 0.50 (0.20–1.0), and 0.30 (0.10–0.50) for the CKD, adult control, and young healthy groups, respectively. The patients with CKD had the highest VI, and there was a statistically significant difference between the groups (p < 0.001) (Figure 4). When patients with CKD were evaluated in subgroups according to the presence or absence of additional disease, no significant difference was found between the groups in VI (p > 0.05).

**Figure 5:**
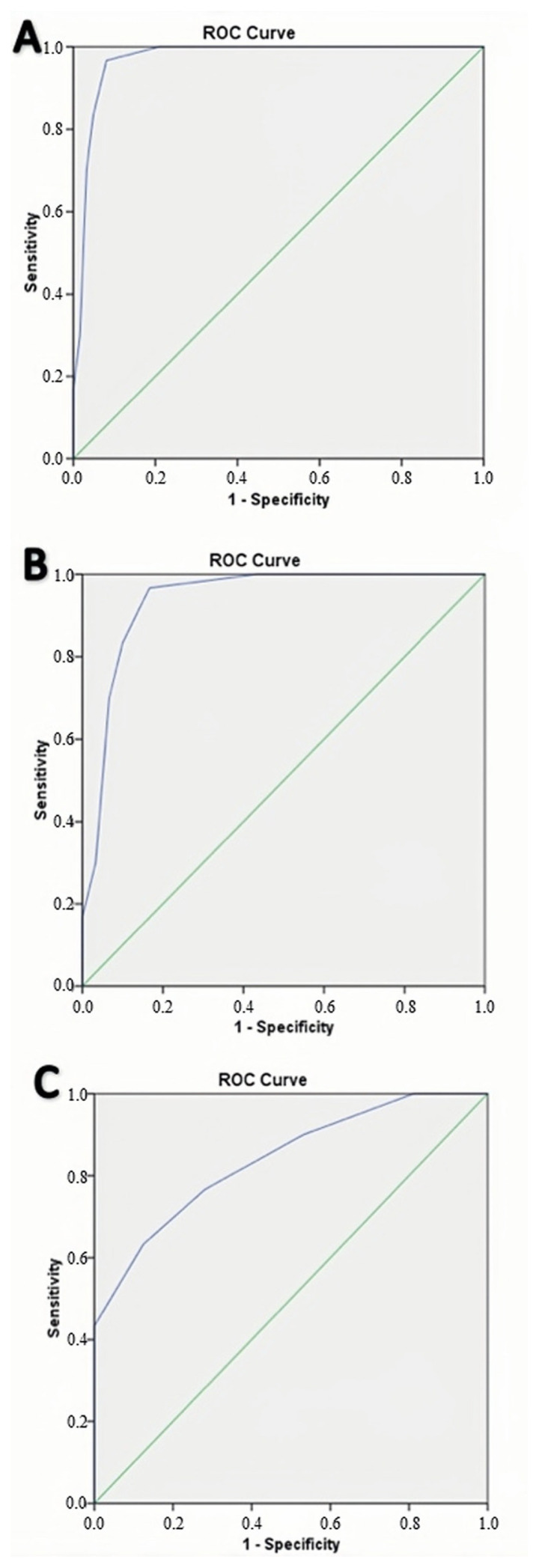
ROC curves with AUC for differentiating CKD patients from adult patients and healthy volunteers. CKD vs. all control group B (A), CKD vs. adult patients group C (B), adult patients group vs. young, healthy group (C).

ROC analysis revealed that RF muscle VI ⊠ 6.5 could distinguish CKD from all groups, the CKD from the adult group without CKD, and adult patients from young healthy individuals with 93.5%, 90.0%, and 74.2% accuracy, respectively (Table 2) (Figures 5A, 5B, and 5C). Moreover, VI demonstrated excellent diagnostic performance in detecting increased blood supply in RF muscle in patients with CKD.

## 4. Discussion

This study has four major findings. First, we found a significant increase in VI values without a decrease in RF-CSA values in patients with CKD. Second, we demonstrated increased vascularity for the adult patient group and patients with CKD by quantifying VI using SMI with objective numerical data. Third, the increase in VI values for patients with CKD was markedly higher than for the adult group. Fourth, we found cut-off values that could be used to detect low-grade inflammation in the RF muscle among patients with CKD and adults using SMI.

When weak muscular strength is detected, MWDs (such as sarcopenia) are likely. A reduced amount or quality of muscle confirms a sarcopenia diagnosis. Sarcopenia is considered severe when there is a combination of reduced muscular strength, low muscle quantity or quality, and poor physical performance. With an increasing need to analyze muscles and diagnose sarcopenia in its early phases, high-resolution imaging is anticipated to be used more frequently in the future, first in research investigations, then in clinical practice [6].

In a recent study to evaluate the anatomic architectural features of the RF muscle in healthy older adults, the mean value of RF-CSA was determined as 4.6 cm^2^ [19]. In another study comparing healthy and older patients affected by chronic obstructive pulmonary disease, RF-CSA was measured as 4.63 cm^2^ and 3.48 cm^2^, respectively [20]. In studies conducted with patients with chronic diseases or hospitalized patients, bioelectrical impedance analysis (BIA) was also evaluated and patients with low BIA values were compared with those with normal values. It has been reported that patients with low BIA values have significantly lower RF-CSA values than other groups [21–23]. Although not statistically significant, RF-CSA values were highest in the healthy young group and lowest in the CKD group in this study, consistent with the literature. We did not use BIA in our study; however, the difference between healthy young people and the other groups was not statistically significant, and all RF-CSA values were similar to healthy groups in the literature. These findings show there is an increase in blood supply in the RF muscle during the period before significant muscle mass loss occurs. We believe this is critical in terms of the effort to obtain quantitative data and cut-off values for the period preceding muscle mass loss, which is also highlighted in the literature [1,3].

Inflammation is the body’s natural defense reaction to foreign pathogens or damage, and it functions to heal tissue and preserve homeostasis. Age-related inflammation is low-grade and ongoing, with increasing proinflammatory cytokines and C-reactive protein levels and a decrease in antiinflammatory cytokines. However, it is asymptomatic with different degrees of pathophysiologic alterations. With age, this alteration in the immune system is known as inflamm-aging or chronic inflammation. It has also been defined as a feature of “immunosenescence” [7]. Chronic inflammation impairs the aging body in various ways (for example, insulin irregularity, hormonal and epigenetic changes, endothelial malfunction, and microvascular alterations). These problems may cause sarcopenia by rendering muscles weaker, speeding up cellular metabolism, and making it harder to keep track of energy. Furthermore, the association between chronic inflammation and muscle wasting has been defined as the influence of the homeostatic equilibrium between protein synthesis and catabolism at the muscular level. Besides, impaired immune homeostasis can contribute to the sarcopenia cascade during the chronic inflammatory state by directly causing the loss of regeneration potential of muscle stem cells [7,24].

Microcirculation is highly sensitive to inflammatory processes and plays an essential role in the inflammatory response. Throughout inflammation, all segments of the microvasculature, such as arterioles, capillaries, and venules, exhibit distinct physiologic alterations that aim to increase the transport of inflammatory cells. One of the most well-known effects of inflammation on microcirculation is an increase in the rate of blood vessel proliferation [10]. Although color DUS and power DUS are commonly used diagnostic tools for evaluating blood flow, they are insufficient for determining microcirculation [17,25]. Therefore, we used SMI, a unique Doppler examination, to assess microcirculation with the adults and patients with CKD in this study, which enabled us to analyze the microcirculatory blood flow in the RF muscles of patients in the study groups together with the differences between the groups.

According to the European Working Group on Sarcopenia in Elderly People, sarcopenia can be primary or secondary. The former is associated with aging, whereas the latter, which occurs in early adulthood, is related to other conditions, and may or may not be related to aging [4,26]. Secondary sarcopenia can occur as a result of nutritional causes such as malnourishment, malabsorption disorders, and anorexic drugs; reduced activity settings such as rest cure, zero-gravity environments, and sedentary lifestyle; and diseases, for example, severe organ failure, inflammatory conditions, and endocrine or malignant diseases. The main distinction between primary and secondary sarcopenia is that muscle loss in the secondary form is age-related. In addition, muscle mass loss also occurs consistently after the fourth decade of life. It also depends on conditions that accelerate protein breakdown and, as a result, is more severe and occurs more often than as a result of the natural aging process [4,26]. When comparing age-related and CKD-related sarcopenia, the most notable distinction is that protein breakdown is evident in CKD-related sarcopenia but may not be the case in age-related sarcopenia. It has also been reported that inflammation is more prominent in CKD-related than in age-related sarcopenia.

We recorded higher VI values in patients with CKD than in both adult and young individuals in our study, supporting the more inflammatory condition revealed by all of these mechanisms. By comparing the groups, we found data demonstrating that inflammation increased with age, and, as stated, increased considerably more among patients with CKD. Even though we came to this conclusion without observing a decrease in muscle area in the adults and patients with CKD, we think that either treatment or physical activities that do not lead to muscle atrophy in patients with an increased blood supply in SMI will be important for preventing morbidity and death from sarcopenia in the future.

It seems sensible to investigate the possibility of regenerative therapeutic approaches for regulating the chronic inflammatory response to treat sarcopenia. Modulation of inflammatory signaling pathways is recognized as the primary therapeutic target, especially with some positive results in animal models, and is attractive for application in human trials [7]. The prominence of modulation of inflammation in treatment strategies also reveals the importance of early recognition of the inflammatory process. This study can also measure how well therapy works, and we hope it will be used as a model for future studies in this area.

As the world’s population over 60 years is predicted to double in the next 30 years, the clinical and economic implications of sarcopenia are becoming a public health issue. The global frequency of sarcopenia is anticipated to increase from 50 million in 2010 to over 200 million by 2050 [1,26]. As a result, detecting sarcopenia early and implementing preventative interventions will save money in the long run by reducing treatment costs and labor losses. SMI, an imaging modality that does not require contrast material, does not include radiation and is easily accessible, will be able to predict sarcopenia among older patients and people with CKD. Therefore, SMI has recently been a popular way to evaluate MSK disorders, particularly inflammatory diseases. Even though US has been used to measure muscle mass in patients with sarcopenia and to assess the structure and elasticity of the muscle sonographically, there have been no studies with SMI evaluating the microvascular system and inflammation during the period before muscle mass loss begins.

There are several limitations to this study. The number of patients in this study was limited, and a larger sample size is needed to validate our results. We did not test for inflammatory indicators in the patients’ serum. However, this may provide an important direction for future studies. We could not assess interobserver variability because a radiologist with SMI experience performed all the examinations. Although monochrome SMI can display more details about vascular architecture [25], we were unable to employ monochrome SMI because VI cannot be quantified using monochrome SMI in current US devices. The patients’ muscular functioning, nutrition, and exercise status were not evaluated because this was a preliminary study, and they did not have a confirmed diagnosis of sarcopenia. The planned next steps for the investigation include involving people who have a clear diagnosis.

In conclusion, SMI can contribute to the detection of low-grade inflammation with quantitative data among adult patients, particularly those with CKD. Due to the constraints of current imaging methods in detecting sarcopenia and the efforts to obtain quantitative data and determine cut-offs, which are the most significant limitations in recent studies, we hope that our research will significantly contribute to mitigating these limitations and lead to further studies in this area.

## Figures and Tables

**Table 1:** Clinical characteristics and ultrasound findings of patients and healthy volunteers.

	Chronic kidney disease (n = 30)	Adult control group (n = 30)	Young healthy group (n = 32)	p-value
**Gender** *				0.578
Male	15 (50)	14 (47)	15 (47)	
Female	15 (50)	16 (53)	17 (53)	
**Age (years)** **	60.17 ± 17.44 b	54.73 ± 9.09 c	30.22 ± 5.31 b,c	**<0.001**
**Duration of the disease (years)**	4.3 ± 2.97	-		-
**Additional disease**				
with	8 (26.7)	-		-
without	22 (73.3)	-		-
**BMI (kg/m^2^)** **	26.33 ± 5.58	26.32 ± 3.32	24.87 ± 2.99	0.273
**Muscle area (cm^2^)** **	4.45 ± 1.67	4.76 ± 1.18	4.99 ± 1.37	0.331
**VI** ***	0.90 (0.60–1.30) a,b	0.50 (0.20–0.10) a,c	0.30 (0.10–0.50) b,c	**<0.001**

* Chi-square tests, data are presented as counts, with percentages in brackets;

** One-Way ANOVA test, data are presented as mean ± standard deviation;

*** Kruskal-Wallis test, data are presented as median (min-max); bold values indicated that statistically significant (p < 0.05);

a Chronic kidney disease vs. adult control group (p < 0.001),

b Chronic kidney disease vs. young healthy group (p < 0.001),

c Adult control group vs. young healthy group (p < 0.001). BMI: Body mass index; VI: Vascular index.

**Table 2:** Diagnostic performance of vascular index (VI) values to distinguish chronic kidney disease (CKD) patients from adult patients and healthy volunteers.

	CKD vs. all control group	CKD vs. adult patients group	Adult patients vs. young healthy group
**AUC (95% CI)**	0.970 (0.938–1.000)	0.939 (0.875–1.000)	0.839 (0.742–0.936)
**p-value**	**<0.001**	**<0.001**	**<0.001**
**Cut-off**	>0.65	>0.65	>0.35
**Sensitivity**	96.7 (82.2–99.9)	96.7 (82.8–99.9)	76.7 (57.7–90.1)
**Specificity**	91.9 (82.2–97.3)	83.3 (65.3–94.4)	71.9 (53.3–86.3)
**PPV**	85.3 (71.4–93.1)	85.3 (72.2–92.8)	71.9 (58.7–82.2)
**NPV**	98.3 (89.2–99.8)	96.2 (78.3–99.4)	76.7 (62.4–86.7)
**Accuracy**	93.5 (86.3–97.6)	90.0 (79.5–96.2)	74.2 (61.5–84.5)

AUC: Area under the curve; 95% CI: 95% confidence interval; bold values indicated that statistically significant (p < .05); PPV: Positive predictive value; NPV: Negative predictive value.

## References

[1] Albano D Messina C Vitale J Sconfienza LM Imaging of sarcopenia: old evidence and new insights European Radiology 2020 30 4 2199 2208 https://doi.org10.1007/s00330-019-06573-2 31834509 10.1007/s00330-019-06573-2

[2] Cruz-Jentoft AJ Landi F Schneider SM Zuniga C Arai H Prevalence of and interventions for sarcopenia in ageing adults: a systematic review. Report of the International Sarcopenia Initiative (EWGSOP and IWGS) Age and Ageing 2014 43 6 748 759 https://doi.org10.1093/ageing/afu115 25241753 10.1093/ageing/afu115 PMC4204661

[3] Sergi G Trevisan C Veronese N Lucato P Manzato E Imaging of sarcopenia European Journal of Radiology 2016 85 8 1519 1524 https://doi.org10.1016/j.ejrad.2016.04.009 27117135 10.1016/j.ejrad.2016.04.009

[4] Sabatino A Cuppari L Stenvinkel P Lindholm B Avesani CM Sarcopenia in chronic kidney disease: what have we learned so far? Journal of Nephrology 2021 34 4 1347 1372 https://doi.org10.1007/s40620-020-00840-y 32876940 10.1007/s40620-020-00840-y PMC8357704

[5] Cruz-Jentoft AJ Baeyens JP Bauer JM Boirie Y Cederholm T Sarcopenia: European consensus on definition and diagnosis: Report of the European Working Group on Sarcopenia in Older People Age and Ageing 2010 39 4 412 423 https://doi.org10.1093/ageing/afq034 20392703 10.1093/ageing/afq034 PMC2886201

[6] Cruz-Jentoft AJ Bahat G Bauer J Boirie Y Bruyere O Sarcopenia: revised European consensus on definition and diagnosis Age and Ageing 2019 48 1 16 31 https://doi.org10.1093/ageing/afy169 30312372 10.1093/ageing/afy169 PMC6322506

[7] Chhetri JK de Souto Barreto P Fougere B Rolland Y Vellas B Cesari M Chronic inflammation and sarcopenia: A regenerative cell therapy perspective Experimental Gerontology 2018 103 115 23 https://doi.org10.1016/j.exger.2017.12.023 29331536 10.1016/j.exger.2017.12.023

[8] Souza VA Oliveira D Barbosa SR Correa J Colugnati FAB Sarcopenia in patients with chronic kidney disease not yet on dialysis: Analysis of the prevalence and associated factors Public Library of Science One 2017 12 4 e0176230 https://doi.org10.1371/journal.pone.0176230 28448584 10.1371/journal.pone.0176230 PMC5407780

[9] Newman JS Adler RS Bude RO Rubin JM Detection of soft-tissue hyperemia: value of power Doppler sonography American Journal of Roentgenology 1994 163 2 385 389 https://doi.org10.2214/ajr.163.2.8037037 8037037 10.2214/ajr.163.2.8037037

[10] Granger D Senchenkova E Inflammation and the Microcirculation Morgan & Claypool Life Sciences San Rafael, CA, USA 2010 21452440

[11] Mourtzakis M Parry S Connolly B Puthucheary Z Skeletal Muscle Ultrasound in Critical Care: A Tool in Need of Translation Annals of the American Thoracic Society 2017 14 10 1495 503 https://doi.org10.1513/AnnalsATS.201612-967PS 28820608 10.1513/AnnalsATS.201612-967PS PMC5718569

[12] Brandenburg JE Eby SF Song P Zhao H Brault JS Ultrasound elastography: the new frontier in direct measurement of muscle stiffness Archives of Physical Medicine and Rehabilitation 2014 95 11 2207 2219 https://doi.org10.1016/j.apmr.2014.07.007 25064780 10.1016/j.apmr.2014.07.007 PMC4254343

[13] Mitchell WK Phillips BE Williams JP Rankin D Smith K Development of a new Sonovue contrast-enhanced ultrasound approach reveals temporal and age-related features of muscle microvascular responses to feeding Physiological Reports 2013 1 5 e00119 https://doi.org10.1002/phy2.119 24303186 10.1002/phy2.119 PMC3841050

[14] Lim AKP Satchithananda K Dick EA Abraham S Cosgrove DO Microflow imaging: New Doppler technology to detect low-grade inflammation in patients with arthritis European Radiology 2018 28 3 1046 1053 https://doi.org10.1007/s00330-017-5016-4 29022101 10.1007/s00330-017-5016-4 PMC5811585

[15] Boote EJ AAPM/RSNA physics tutorial for residents: topics in US: Doppler US techniques: concepts of blood flow detection and flow dynamics Radiographics 2003 23 5 1315 1327 https://doi.org10.1148/rg.235035080 12975518 10.1148/rg.235035080

[16] Gitto S Messina C Chianca V Tuscano B Lazzara A Superb microvascular imaging (SMI) in the evaluation of musculoskeletal disorders: a systematic review La Radiologia Medica 2020 125 5 481 490 https://doi.org10.1007/s11547-020-01141-x 32020529 10.1007/s11547-020-01141-x

[17] Samanci C Ozkose B Ustabasioglu FE Erol BC Sirolu S The Diagnostic Value of Superb Microvascular Imaging in Prediction of Uterine Artery Embolization Treatment Response in Uterine Leiomyomas Journal of Ultrasound in Medicine 2021 40 12 2607 2615 https://doi.org10.1002/jum.15647 33599335 10.1002/jum.15647

[18] Deng M Liang C Yin Y Shu J Zhou X Ultrasound assessment of the rectus femoris in patients with chronic obstructive pulmonary disease predicts poor exercise tolerance: an exploratory study BMC Pulmonary Medicine 2021 21 1 304 https://doi.org10.1186/s12890-021-01663-8 34563152 10.1186/s12890-021-01663-8 PMC8466975

[19] El-Ansary D Marshall CJ Farragher J Annoni R Schwank A Architectural anatomy of the quadriceps and the relationship with muscle strength: An observational study utilising real-time ultrasound in healthy adults Journal of Anatomy 2021 239 4 847 855 https://doi.org10.1111/joa.13497 34458993 10.1111/joa.13497 PMC8450473

[20] Seymour JM Ward K Sidhu PS Puthucheary Z Steier J Ultrasound measurement of rectus femoris cross-sectional area and the relationship with quadriceps strength in COPD Thorax 2009 64 5 418 423 https://doi.org10.1136/thx.2008.103986 19158125 10.1136/thx.2008.103986

[21] Matsuzawa R Yamamoto S Suzuki Y Imamura K Harada M The clinical applicability of ultrasound technique for diagnosis of sarcopenia in hemodialysis patients Clinical Nutrition 2021 40 3 1161 1167 https://doi.org10.1016/j.clnu.2020.07.025 32798065 10.1016/j.clnu.2020.07.025

[22] Eşme M Karcıoğlu O Öncel A Ayçiçek GŞ Deniz O Ultrasound Assessment of Sarcopenia in Patients With Sarcoidosis Journal of Ultrasound in Medicine 2022 41 4 951 959 https://doi.org10.1002/jum.15780 34268780 10.1002/jum.15780

[23] Ozturk Y Koca M Burkuk S Unsal P Dikmeer A The role of muscle ultrasound to predict sarcopenia Nutrition 2022 101 111692 https://doi.org10.1016/j.nut.2022.111692 35660496 10.1016/j.nut.2022.111692

[24] Han A Bokshan SL Marcaccio SE DePasse JM Daniels AH Diagnostic Criteria and Clinical Outcomes in Sarcopenia Research: A Literature Review Journal of Clinical Medicine 2018 7 4 70 https://doi.org10.3390/jcm7040070 29642478 10.3390/jcm7040070 PMC5920444

[25] Jo E Lee SR Park BS Kim JS Potential mechanisms underlying the role of chronic inflammation in age-related muscle wasting Aging Clinical and Experimental Research 2012 24 5 412 422 https://doi.org10.3275/8464 22717404 10.3275/8464

[26] Durmaz MS Sivri M Comparison of superb micro-vascular imaging (SMI) and conventional Doppler imaging techniques for evaluating testicular blood flow Journal of Medical Ultrasonics 2018 45 3 443 452 https://doi.org10.1007/s10396-017-0847-9 29248966 10.1007/s10396-017-0847-9

